# Identification of High Nitrogen Use Efficiency Phenotype in Rice (*Oryza sativa* L.*)* Through Entire Growth Duration by Unmanned Aerial Vehicle Multispectral Imagery

**DOI:** 10.3389/fpls.2021.740414

**Published:** 2021-12-03

**Authors:** Ting Liang, Bo Duan, Xiaoyun Luo, Yi Ma, Zhengqing Yuan, Renshan Zhu, Yi Peng, Yan Gong, Shenghui Fang, Xianting Wu

**Affiliations:** ^1^State Key Laboratory of Hybrid Rice, Wuhan University, Wuhan, China; ^2^College of Life Sciences, Wuhan University, Wuhan, China; ^3^Lab of Remote Sensing for Crop Phenomics, Wuhan University, Wuhan, China; ^4^Oil Crops Research Institute, Chinese Academy of Agricultural Sciences, Wuhan, China; ^5^School of Remote Sensing and Information Engineering, Wuhan University, Wuhan, China; ^6^Crop Research Institute, Sichuan Academy of Agricultural Sciences, Chengdu, China

**Keywords:** UAV, high throughput phenotyping, entire growth duration, canopy nitrogen content, high NUE phenotype

## Abstract

Identification of high Nitrogen Use Efficiency (NUE) phenotypes has been a long-standing challenge in breeding rice and sustainable agriculture to reduce the costs of nitrogen (N) fertilizers. There are two main challenges: (1) high NUE genetic sources are biologically scarce and (2) on the technical side, few easy, non-destructive, and reliable methodologies are available to evaluate plant N variations through the entire growth duration (GD). To overcome the challenges, we captured a unique higher NUE phenotype in rice as a dynamic time-series N variation curve through the entire GD analysis by canopy reflectance data collected by Unmanned Aerial Vehicle Remote Sensing Platform (UAV-RSP) for the first time. LY9348 was a high NUE rice variety with high Nitrogen Uptake Efficiency (NUpE) and high Nitrogen Utilization Efficiency (NUtE) shown in nitrogen dosage field analysis. Its canopy nitrogen content (CNC) was analyzed by the high-throughput UAV-RSP to screen two mixed categories (51 versus 42 varieties) selected from representative higher NUE *indica* rice collections. Five Vegetation Indices (VIs) were compared, and the Normalized Difference Red Edge Index (NDRE) showed the highest correlation with CNC (*r* = 0.80). Six key developmental stages of rice varieties were compared from transplantation to maturation, and the high NUE phenotype of LY9348 was shown as a dynamic N accumulation curve, where it was moderately high during the vegetative developmental stages but considerably higher in the reproductive developmental stages with a slower reduction rate. CNC curves of different rice varieties were analyzed to construct two non-linear regression models between N% or N% × leaf area index (LAI) with NDRE separately. Both models could determine the specific phenotype with the coefficient of determination (*R*^2^) above 0.61 (Model I) and 0.86 (Model II). Parameters influencing the correlation accuracy between NDRE and N% were found to be better by removing the tillering stage data, separating the short and long GD varieties for the analysis and adding canopy structures, such as LAI, into consideration. The high NUE phenotype of LY9348 could be traced and reidentified across different years, locations, and genetic germplasm groups. Therefore, an effective and reliable high-throughput method was proposed for assisting the selection of the high NUE breeding phenotype.

## Introduction

Nitrogen (N) is one of the essential nutrients for plants and plays a central role in photosynthesis, energy transmission, morphological construction, and biomass synthesis (Xu et al., 2012). Nitrogen is absorbed from the soil, transported through the vascular bundles, assimilated into N-containing organic compounds, decomposed, and remobilized to maintain a balance in the nutrients within plants. This is regulated by a systematic network of genes at multiple levels that are involved in many important physiological pathways, such as carbon metabolism (Evans, 1989; Robertson and Vitousek, 2009; Dechorgnat et al., 2011; Kant et al., 2011; Xu et al., 2012; Hu and Zhang, 2014; Han et al., 2015; Sun et al., 2016; Li et al., 2017; Xuan et al., 2017; Perchlik and Tegeder, 2018; Wang Y. et al., 2018; Xin et al., 2019). Therefore, overcoming the soil N limitation by chemical N application is crucial to increase the harvest index (HI) and yield for field management (Robertson and Vitousek, 2009; Lassaletta et al., 2016; Liang et al., 2017).

In the past 6 decades, global N fertilizer usage increased more than ten times from 11.3 Tg/year in 1961 to 118.7 Tg/year in 2020 to produce food for the rapidly growing population (Swarbreck et al., 2019). In China, cereal grain yield increased by 65% due to an increase in N fertilizer consumption by 512% from 1980 to 2010 (Chen et al., 2011; Zhang et al., 2011). The cost of damages caused by excess N application is estimated to be US$91–466 billion per year in Europe (Han et al., 2015). However, the increase in yield is not proportional to the increase in N fertilizer application, and a plateau has been reached (Ju et al., 2009; Wang and Peng, 2017). Higher N input causes lower Nitrogen Use Efficiency (NUE) because of the rapid N loss by denitrification, volatilization, surface runoff, and leaching into the soil and water, resulting in serious environmental problems (Robertson and Vitousek, 2009). Fertilizer costs worth US$2.3 billion can be saved annually if the Nitrogen Uptake Efficiency (NUpE) is increased by 1% (Raun and Johnson, 1999).

Rice (*Oryza sativa* L.) is a staple food crop for almost 50% of the world population. Rice production in China is the highest in the world. Although the average N input in the paddy fields of China is the highest between 180–209 kg/ha (world average: 105 kg/ha) (FAO, 2015; Guo et al., 2017), the efficiency of N usage is only approximately 30–35% (Gu et al., 2017; Guo et al., 2017; Wang and Peng, 2017). Optimizing N fertilizer management has been performed to significantly increase NUE in rice production and reduce N input to 150–165 kg/ha (Li et al., 2012; Liu et al., 2013; Wang and Peng, 2017). However, to reach the maximum yield potential at 10–15 Mt/ha, N inputs for most super rice varieties have to be more than 300 kg/ha (Li et al., 2012; Sun et al., 2013; Wei et al., 2016; Liang et al., 2017). Therefore, only relying on N fertilizer cannot be a fundamental solution to improve cultivation. The selection of high-yielding rice varieties with a higher NUE at a lower N input level is the key strategy for cost-effectiveness. However, a lack of known reliable high NUE phenotypes is the main barrier to the selection of high NUE rice varieties during the breeding and sustainable development of agriculture (Bueren and Struik, 2017).

The main goal of the high NUE studies is to screen for both the high NUpE and Nitrogen Utilization Efficiency (NUtE) phenotypes. The focus of the NUpE phenotype is the selection of the rice varieties with higher yields and reduced exogenous N input, which is relatively easier to perform by controlling N fertilizer dosages for field screening. However, identifying NUtE phenotypes is aimed to increase the efficiency of endogenous N assimilation and transformation within plants, which is regulated by a complicated gene network and balanced at multiple levels of metabolism. Therefore, significant progress has been made in the NUpE studies, while the NUtE screening progress has been relatively slower. The main challenges are the following: (1) Sources of high NUE varieties, especially high NUtE varieties, are rare. Super rice and green super rice breeding programs in China were proposed to select high yield NUE rice varieties (Wang and Peng, 2017; Huang et al., 2018). However, most of the varieties tested were mainly from the Wild Abortive Cytoplasmic Male Sterility (WA-CMS) germplasm. Different from the WA-CMS and Baotai CMS (BT-CMS) varieties, many varieties with green phenotypes, such as high NUE (both NUpE and NUtE) and broader biocompatibility, were generated from the Honglian type CMS (HL-CMS) variety (Li and Zhu, 1988; Liu et al., 2004; Wu et al., 2016). Therefore, it is worth characterizing the high NUE phenotypes of these varieties, such as LY9348. (2) High NUE phenotypes are hard to define and trace since canopy nitrogen content (CNC) or leaf nitrogen content (LNC) change with the field N supply levels, root absorption and transportation abilities, different developmental stages, and genetic variation (Li et al., 2017; Lian et al., 2019; Marshall-Colon and Kliebenstein, 2019; Watt et al., 2020). Difficulties in phenotype identification may have been one of the reasons for not cloning NUE genes by map-based cloning methods so far (Good et al., 2004; Han et al., 2015). (3) High NUE phenotypes are compound agricultural traits with complicated gene network regulations (Liang et al., 2021). Using Marker-Assisted Selection (MAS) at the molecular level based on one or a few genes does not provide enough support to reliably select phenotypes for breeding purposes. Gene chips, single nucleotide polymorphism (SNP) assisted high-throughput selection can provide broader gene information. However, high NUE genes are not well-defined to support a genome-wide selection of molecular markers (Yang et al., 2014; Sandhu et al., 2019; Tang et al., 2019). Lastly, (4) High NUE phenotypes are hard to trace less laboriously in real-time by using traditional, non-destructive, and sensitive techniques (Wang J. et al., 2019; Bacenetti et al., 2020). Thus, new techniques should be explored.

Remote sensing (RS), as a non-destructive measurement technique, has an enormous potential to determine NUE phenotypes. RS can efficiently obtain vegetation spectral data, which contains a lot of information on the interactions between vegetation and solar radiation (Thenkabail et al., 2011). It has been shown that leaf pigments strongly absorb visible light, thus reducing the reflectance of vegetation in the visible range (Woolley, 1971). Therefore, many optical devices have been developed to estimate the pigment content based on the RS mechanism. Additionally, some handheld devices, such as the Soil Plant Analysis Development (SPAD)-502 chlorophyll meter and the N-pen nitrogen meter, are used to obtain leaf reflectance data for determining the leaf pigment content (Liu et al., 2017). At the close-range canopy level (1 m above the canopy), the Analytical Spectral Devices (ASD) Field Spec 4 spectrometer is often used to collect canopy reflectance in the visible and near-infrared range, and the vegetation index calculated from the reflectance of the different spectral bands is used to estimate the pigment content (Schlemmer et al., 2013; Ge et al., 2019; Li et al., 2019). Although the leaf-level and canopy-level devices can estimate pigment content non-destructively, it is still hard to implement them for large-scale measurements in field breeding practices. Recently, the Unmanned Aerial Vehicle Remote Sensing Platform (UAV-RSP) with multispectral sensors has become an easy operational base for high-throughput field phenotyping (HFP) in large-scale field studies (Yang et al., 2017), which can be used to discover dynamic novel traits invisible to the human eye, such as leaf area index (LAI), N accumulation in the canopy, drought adaptive traits, and yield estimation (Kyratzis et al., 2017; Condorelli et al., 2018; Zheng et al., 2018; Blancon et al., 2019; Duan et al., 2019a,b; Prey et al., 2020).

Unmanned Aerial Vehicle Remote Sensing Platform (UAV-RSP) becomes an increasingly significant technology in the industry 4.0 applications supporting multiple devices to build the Internet of Things (IoT) network in the 5G era and beyond 5G (B5G) (Syed et al., 2020; Alsamhi et al., 2021). Great achievements in crop monitoring are constructed between UAV-RSP, Wireless Sensor Networks (WSNs), and IoT. These efforts would give far-reaching consequences on UAV-WSN-IoT roles in smart farming and precision agriculture system construction (Saif et al., 2017; Almalki et al., 2021). In addition, with the development of artificial intelligence, UAV-RSP can take advantage of high throughput to monitor the nutrient level, plant disease, and insect pest automatically (Freitas et al., 2020; Tetila et al., 2020; Lpo et al., 2021). Therefore, UAV-RSP, coupled with IoT and 5G technology, plays a more and more important role in building the intelligent monitoring network for smart inspection of plant growth status (Syed et al., 2020; Almalki et al., 2021). However, the application in breeding selections is seldomly explored due to the complexity of plant phenotype extraction and lack of growth models for important agricultural traits, which is the key challenge for the seeds industry and modern agriculture. To our best knowledge, this is the first comprehensive research to extract NUE phenotype in rice as a dynamic time-series N variation curve through the entire GD analysis by canopy reflectance data collected by UAV-RSP, especially for NUE phenotypes identification and construction of N smart inspection models.

In the past decades, LNC was measured non-destructively by RS using linear or non-linear models constructed to establish relationships between spectral features and CNC/LNC (Li D. et al., 2018). The Shortwave infrared region (SWIR, 1,000–2,400 nm) can accurately estimate LNC from dried leaves but not fresh leaves. The Visible and near-infrared region (VNIR, 400–1,000 nm) can be better correlated with chlorophyll and N in fresh leaves (Afandi et al., 2016; Li D. et al., 2018). Vegetation Indices (VIs), such as the Normalized Difference Vegetation Index (NDVI), the Normalized Difference Red Edge Index (NDRE), the Red Edge Chlorophyll Index (CI_rededge_), and the Green Chlorophyll Index (CI_green_), have been used for chlorophyll and N estimations in maize, wheat, and rice (Fitzgerald et al., 2010; Shiratsuchi et al., 2011; Cao et al., 2013; Schlemmer et al., 2013; Inoue et al., 2016; Klem et al., 2018; Wen et al., 2018). However, the accuracy of estimation is influenced by soil backgrounds, genetic differences, and climate change, and varies across seasons and locations. The NDVI is a good index to measure wheat NUE above the canopy at 0.8 m (Klem et al., 2018). The near-infrared band index, such as the Red Edge Spectral Index (REDVI), is a good estimator of the rice N nutrition index (NNI) using a multispectral sensor, collected from < 1 m above the canopy (Cao et al., 2013). Based on UAV multispectral imagery, the Normalized Difference Texture Index (NDTI) was found to be strongly correlated with plant N content (PNC) (Zheng et al., 2018), while the NDRE showed a good relationship with leaf N accumulation (LNA) and plant N accumulation (PNA), but not with LNC and PNC (Zheng et al., 2020). These studies focused on specific growth stages such as booting or grain filling stages by comparing among a few or only between two rice cultivars. Thus, applying these during field screening is difficult as the challenge for NUE phenotype screening is to evaluate N variations in multiple varieties across the entire growth duration (GD).

To apply UAV-RSP at the HFP level for the selection of higher NUE phenotypes, several aspects should be considered: (1) Dynamic N accumulation across the entire GD is critical for the identification of high NUE phenotypes, which could be novel phenotypes, distinguishing higher NUE rice varieties from normal or lower NUE varieties; (2) GD is an important factor influencing many important agricultural parameters such as panicle initiation, flowering time, yield, and biomass (Li F. et al., 2018; Liu et al., 2020; Won et al., 2020). Therefore, when many rice varieties are tested at the high-throughput level, the influence of GD on N measurement needs to be addressed from an agricultural standpoint; (3) The phenotypes identified should be stable and traceable across years and locations to make reliable decisions while screening; (4) CNC measurements of hundreds or thousands of rice varieties can be performed in a uniform background of soil and climate. Therefore, besides selecting VIs, the parameters that influence the accuracy of N inspection can be compared; (5) Phenotypes can be easily analyzed by breeders to make reliable decisions while characterizing higher NUE varieties. To find solutions for these aspects, this study aimed to use a high NUE *Oryza sativa indica* rice variety LY9348, mixed with two different *O. sativa indica* groups (Li et al., 2014) (NUE is higher in *O. sativa indica* than in *O. sativa japonica* rice) (Hu et al., 2015), to test the following hypotheses: (1) CNC changes in LY9348 through the entire GD can be detected as a dynamic but specific N accumulation phenotype for assisting high NUE breeding selections; (2) GD differences among rice varieties may impact the accuracy of N estimation; (3) Factors such as canopy structures, genetic variations, and developmental stages may influence N estimation accuracy; (4) This phenotype can be traced using the UAV-RSP for breeders to select plants with high NUE in mixed field trials; (5) The cost can be lowered if the plot size is reduced to assist large-scale or super-large-scale selection of high NUE phenotypes at the high-throughput level.

## Materials and Methods

### Field Nitrogen Dosage Analysis for the Evaluation of Nitrogen Use Efficiency in LY9348

Plant material information was listed in Experiment 1 (Table 1). Previous field trials had shown that LY9348 had a similar yield to that of the Huanghuazhan rice variety (a popular variety cultivated in the southern regions of China) with 25% less N input (data not shown). Based on the previous result, LY9348 and FLY4H (a standard high yield control (CK) for the National Rice Variety Regional Test in China) were planted and treated with one of four nitrogen dosages (N_0 =_ 0 kg/ha, N_8 =_ 120 kg/ha, N_12 =_ 180 kg/ha, and N_16 =_ 240 kg/ha). LY9348 and FLY4H were planted adjacent to one another in one experimental block for each dosage of nitrogen (Supplementary Figure 1). Each plot was approximately 30 m^2^ (10 m × 3 m) and divided into two halves for LY9348 and FLY4 (15 m^2^, 5 m × 3 m for each half). Margin balks (0.4 m wide) were built and covered by plastic films during the entire experiment to avoid N leaching or permeating into the other experimental blocks (Supplementary Figure 1A). Three blocks for each dosage analysis were arranged randomly as replicates in the field for a total of 12 experimental blocks (Supplementary Figure 1B).

**TABLE 1:** The details of the four experiments conducted in Hainan and Hubei from 2017 to 2018.

	Sowing date and transplanting date	Location	Number of rice varieties	Plant number and size of one plot	Altitude of UAV flight	Field measurement	Data analysis
Experiment 1	December 10th, 2017 January 6th, 2018	Hainan	2	864 plants 10 m × 3 m	—	PL, EPN, GNP, SSR, TGW, GYP, GY	Yield traits
Experiment 2	May 10th,2017 May 31st,2017	Hubei	51	60 plants 1.2 m × 1.6 m	50 m	SPAD, N%_N–Pen_, N%_EQA_, ASD	GD analysis
Experiment 3	December 10th, 2017 January 6th, 2018	Hainan	51	60 plants 1.2 m × 1.6 m	50 m	SPAD, N%_N–Pen_, N%_EQA_	Model I
Experiment 4	December 10th, 2017 January 5th, 2018	Hainan	42	1500 plants 6.0 m × 11.0 m	200 m	N%_EQA_, LAI	Model II

*PL, panicle length; EPN, effective panicle number; GNP, grain number per panicle; SSR, seeds setting rate; TGW, thousand grains weight; GYP, grain yield per plant; GY, grain yield; N%_EQA_, nitrogen content measured by elementary quantitative analysis (EQA); SPAD, SPAD value measured by SPAD-502 chlorophyll meter; N%_N–Pen_, nitrogen content measured by N-pen meter; ASD, close-range canopy reflectance collection by ASD Field Spec 4 spectrometer; LAI, leaf area index; GD, growth duration.*

In each half block, 432 individual plants of each variety were arranged at a hill spacing of 15 cm × 18 cm in 24 rows with 18 plants per row, occupying the 15 m^2^ field space. Superphosphate (90 kg/ha P_2_O_5_) and potassium sulfate (180 kg/ha K_2_O) were applied as basal fertilizers in all blocks before transplanting. Urea (N) applied for each dosage was divided into three parts to be applied in the seedling, tillering, and booting stages separately to each experimental block. After transplanting, 5 cm water depth was maintained in each block. Water maintained in the plot was drained for ten days before the harvest to facilitate harvesting. Insects, diseases, and weeds were intensively controlled to avoid yield loss, besides the regular field management practices.

Yield-related phenotypes were statistically characterized. If one panicle contained more than five full grains, the panicle was counted as one effective panicle. All panicles from the main shoot and all tillers were summed to obtain the effective panicle number (EPN) value of each plant. Panicle length (PL) was measured based on the length of the effective panicles from the bottom node to the top tip. All full grains were counted and divided by the total EPNs of each plant to calculate the grain number per panicle (GNP). From each plant, 200 grains were weighed, and the value was multiplied by 5 to obtain the value of the thousand grains weight (TGW). The seed setting rate (SSR) per panicle was calculated by the following equation: (number of full grains/number of total grains) × 100%. All full grains were weighed to obtain the grain yield per plant (GYP). All the yield-related parameters were calculated by the average value of 30 individual plants (10 plants randomly chosen from each dosage plot and three replicate plots for each dosage treatment). Grain yield (GY, kg/ha) was obtained based on 100 harvested plants from the equation: GY = [(GYP × plants counted)/field area occupied by plants counted in m^2^] × 10,000 (1 hectare = 10,000 m^2^). Grain yield per kg N (NUE) was estimated based on the grain yield from 100 harvested plants from the central area of each plot and adjusted by deducting 13.5% standard moisture content for the calculation. NUE was determined by the equation: NUE = NUpE × NUtE = grain weight gained/soil nitrogen amount supplied (Xu et al., 2012).

### Plant Materials for the Nitrogen Use Efficiency Dynamic Curve Analysis

Information on plant materials was listed in Experiments 2, 3, and 4 (Table 1). Fifty-one rice varieties were planted in scheduled fields in the Wuhan University Rice Experiment and Research Base, Ezhou, Hubei Province, China (30°22’31.4N, 114°44’50.6E) and the Wuhan University Hybrid Rice Experiment and Research Base, Lingshui, Hainan Province, China (18°31’47.1N, 110°03’34.9E). They were selected from the 3000 rice genome project (Li et al., 2014), which is a representative global rice germplasm collection (Supplementary Table 1). Li et al., had classified 3,000 rice accessions into five groups (*japonica*, *indica*, *aus/boro*, *basmati/sadri*, and *intermediate*). N accumulations in the *indica* and the intermediate groups were found to be more efficient than that in the other three groups, and N accumulation in the *aus/boro* group was in between (Hu et al., 2015). Therefore, for the NUE analysis, 51 varieties, including LY9348, were selected from the classified *indica* and *intermediate* groups, including two *aus/boro* varieties, for comparisons to form the experimental test collection. The GD of the varieties among this collection varied and was divided into longer and shorter duration groups for the GD influence analysis.

Forty-two varieties were planted in one field in Lingshui, Hainan, China. To select the 42 varieties (Supplementary Table 2), two main aspects were considered: (1) Many varieties from the 3,000 rice genome project were germplasm collections and not used in field practices because of their lower-yielding, varied GD, and/or weak agricultural traits. Therefore, LY9348 was mixed with currently cultivated rice varieties for evaluation. In addition, two higher-yielding cultivated *Japonica* rice varieties were also included for comparison; (2) LY9348 was the hybrid rice generated from the Honglian type male sterile line LH4A as the female donor and CH9348 as the male donor. Thus, many other Honglian-type hybrid rice varieties were added to the group to exclude the effects of a homogenous background for the identification of the high NUE phenotypes.

The total rice growth period was divided into six typical phenotypic stages as tillering stage (TS), jointing stage (JS), panicle initiation stage (PIS), booting stage (BS), full heading stage (FHS), and milk ripen stage (MRS). For the 51 rice varieties with six developmental stages, all average N values were recorded as 306 data points for each experiment. Similarly, for the 42 rice varieties with six developmental stages, all average N values were recorded as 252 data points in this study.

### Field Management

Planting densities in Experiments 2, 3, and 4 were arranged in two planting modes: (1) Small plot size trials in Ezhou, Hubei (2017) and in Lingshui, Hainan (2018), where 60 plants of each rice variety were transplanted in six lines with 20 cm line spacing, and 10 plants in each line were planted with 16 cm row spacing (1.6 m × 1.2 m). One empty line was maintained between every six-line plot for the ease of identification of the different varieties and processing of the UAV data (1.8 m × 1.2 m); 2) Large block size in Lingshui, Hainan (2018), where 1,500 plants were planted in 1/10 Chinese mu (equals to 66.67 m^2^), which was a standard plant density maintained in the field with lesser than 2,25,000 plants per hectare (average 22.5 plants per square meter, *d* = 22.5) (Table 1).

The total growth period from sowing to the maturation of seeds was between 6 and 7 months, depending on differences in the varieties. Compound fertilizer (375 kg/ha; the ratio of N, P, and K was 15% each) was applied uniformly across the field. Regular rice field management was performed by a field manager. During each experiment, one UAV flight was arranged to obtain the images of all the rice plots, and each plot was scheduled to be measured five times repeatedly to get an average value for statistical analysis. After the UAV flight (from 10 a.m. to 2 p.m.), the corresponding ground measurements were performed immediately *in situ*.

### Nitrogen Content Measurement by Elementary Quantitative Analysis

To quantify the N-uptake ability of LY9348, leaves of LY9348, and those of its parents (LH4B, CH9348) and the other 48 rice varieties were analyzed for N accumulation in six key growth stages. At each developmental stage, leaf samples were collected from each rice variety for accurate N measurements.

In the early developmental stages, before the flag leaf emerged, the fully spread leaf from the top of each plant was collected to measure the N content. In the later stages, after the flag leaf had emerged, the second leaf (when counting downwards from the flag leaf), defined as the functional leaf in rice, was selected for the N measurement of each plant. Three individual plants were selected randomly from each block to collect leaves for the analysis. Leaves were dried at 80°C in an oven (AFD-270L-200, AoFeiDa Instrument and Equipment Co., Ltd., China) for hours until the dry weight was stable. Dry leaf samples were mechanically ground by a ball milling machine (JXFSTPRP-576, Jingxin Instrument, and Equipment Co., Ltd., China), and a fine powder was obtained by filtering through a 100-mesh sieve (φ100 mm, 2.36–0.038 mm, JinHe Machinery Co., Ltd., China). The nitrogen content was measured by a Stable Isotope Ratio Mass Spectrometer (IsoPrime100 IRMS, Isoprime Ltd., United Kingdom) following the protocol provided in a previous study (Sun et al., 2016). Data from each leaf was recorded as one LNC value for that plant, and the average LNC data of three plants was recorded as the LNC value for each rice variety at each stage.

### N-pen Meter Measurements

As an instrument that uses optical methods for the estimation of N levels in leaves, the N-pen meter [Photon Systems Instruments (PSI), spol.s.r.o, Czechia] was used to measure the reflectance of the 51 rice varieties to obtain the Normalized Difference Greenness Index (NDGI). The nitrogen content of each rice variety was measured as reported in maize, wheat, and barley, and the nitrogen content was calculated from a correlation with the NDGI (Klem, 2008). Since the N-pen meter measures the N levels non-destructively, measurements from the same leaf could be taken multiple times, and the optical data could be collected before the leaf was removed for the Elementary Quantitative Analysis (EQA) analysis. Each selected leaf was measured repeatedly six times, and the average value from these six measurements was recorded as the N value of the plant. The average value of three individual plants was recorded as the final N value and displayed in percentage (60 plants/plot). Data transfer and many additional features for data presentation in tables and graphs were processed by the Comprehensive FluorPen 1.1 software (Photon Systems Instruments, spol.s.r.o., Czechia).

### Soil Plant Analysis Development Measurements

Chlorophyll contents were measured by a SPAD-502 Chlorophyll Meter (SPAD-502 Plus, Konica Minolta, Japan), and SPAD values were recorded. Since chlorophyll content is closely related to N content (Ercoli et al., 1993; Baret and Fourty, 1997), SPAD is used to estimate N in agricultural practices, besides the N-pen meter. Therefore, SPAD was used as one of the leaf level N estimation optical methods in this study. Since SPAD measures the chlorophyll content non-destructively, measurements from the same leaf could be taken multiple times, and the optical data could be collected before the leaf was removed for the EQA analysis. Each selected leaf was measured repeatedly five times, the average value of these measurements was recorded as the value for the plant, and the average value of three individual plants was recorded as the final SPAD value (60 plants/plot).

### Close-Range Canopy Reflectance Measurements

The close-range canopy reflectance (350–2,500 nm) of rice was measured by an ASD Field Spec 4 spectrometer (Analytical Spectral Devices Inc., Boulder, CO, United States). Data were collected at 1 m vertical height exactly above the plant canopy every five days when the weather was sunny and clear between 10 am and 2 pm. Each plot was measured repeatedly five times, and the average value of these five measurements was recorded as the canopy reflectance of the plot. Instrumental noise that affected the actual spectral measurements caused by weather variations was reduced by using a standard whiteboard during calibration. The waveband spectral data between 1,301 and 2,500 nm (approximately) was removed from the analysis because of the low level of signal-to-noise ratio (SNR) within this range.

### Leaf Area Index

Five plants were selected randomly from the plots of each variety of rice at every developmental stage (the 42-variety group, 1,500 plants/plot). For each plant, all the leaves were examined before measurements. If more than 50% of a leaf was yellow, the leaf was designated as a yellow leaf. If a plant had more than 50% of yellow leaves, it was not selected for further analysis. Since this study used a destructive method to measure rice materials and multiple developmental stages were considered for the tests, two individual plants with many green leaves were chosen from the five selected plants as representative samples of each rice variety and for each stage. The selected plants, including all tillers, were dug out from the soil along with their roots. These plants were placed in a plastic bucket with a water supply and taken back to the laboratory for analysis after collecting all the samples. All green leaves from the two plants of each rice variety were plucked from the stem for scanning by a Leaf Area Meter LI-3100C (LI-COR Corporate, Lincoln, NE, United States). The scanning DPI was approximately between 0.1 and 1 mm^2^. The average area of all the leaves of the two plants was used as a representative value of the single plant leaf area (LA_S_) of the rice plot. From the plant density in one square meter of each rice variety (*d* = 22.5), the LAI value of each plot was calculated from the equation: LAI = LA_S_ × d.

### Unmanned Aerial Vehicle Data Collection and Processing

To collect rice canopy level reflectance, images of target research plots were taken using a Mini-multiple camera array (MCA) system mounted on a UAV (S1000, SZ DJI Technology, Co., Ltd., China) every 5–7 days depending on sunlight conditions after the rice plants were transplanted until maturation. The Mini-MCA system consisted of an array of twelve individual miniature digital cameras (Mini-MCA 12, Tetracam, Inc., Chatsworth, CA, United States). Each sensor channel could produce 10-bit super extended graphics array (SXGA) data. Each camera imager was equipped with a customer-specified bandpass filter centered at a wavelength of 490, 520, 550, 570, 670, 680, 700, 720, 800, 850, 900, and 950 nm, which are bands commonly used for the analysis of VIs.

The MCA system was attached to the UAV on a gimbal to avoid any unwanted effects caused by the movement of the UAV, and the camera misregistration effect was controlled through the co-registration by12 cameras before the flight, as previously described (Duan et al., 2019a). All UAV flights were performed under clear sky conditions with little cloud cover between 10 am and 2 pm local time, when the changes in the solar zenith angle were minimal at the location of the experiment. For the experiments with the 51 rice varieties, the altitude of UAV flight was 50 m above the target plots at a spatial resolution of 2.7 cm around. For the experiments with the 42 rice varieties, the altitude of UAV flight was 200 m above the target plots at a spatial resolution of 10.8 cm around.

An empirical linear correction method was used to transform the Digital Numbers (DN) of the images into surface reflectance (ρ_λ_) as previously described (Duan et al., 2019a,b). The imaging radiometric correction was modified by a standard consisting of six calibration ground targets, which were placed in the field of view of the cameras before each flight. The research plots and all calibration ground targets were included in the image taken from the Mini-MCA system. In this study, the calibration targets provided relatively stable reflectance of 0.03, 0.12, 0.24, 0.36, 0.56, and 0.80 from the visible wavelength to the near-infrared (NIR) wavelength, respectively, which is commonly applied for the radiometric calibration of aerial images. Since a linear relationship was assumed between DN and ρ_λ_, the rice variety reflectance equation used was


(1) ρλ=DNλ×Gainλ+Offsetλ(λ=490,520,550,570,670,680,700,720,800,850,900,and 950nm)

In this study, ρ_λ_ and DN_λ_ are the surface reflectance and the digital numbers of the image of a given pixel at wavelength λ; Gain_λ_ and Offset_λ_ are camera gains and bias at different wavelengths. For each wavelength, Gain_λ_ and Offset_λ_ were calculated by the method of least squares from ρ andDN values (referring to DN_.03_, DN_.12_, DN_.24_, DN_.36_, DN_.56,_ and DN_.80_) of six calibration targets.


(2) $$\left[\begin{array}{c} \begin{array}{c} 0.03\\ \begin{array}{c} 0.12\\ 0.24 \end{array} \end{array}\\ \begin{array}{c} 0.36\\ \begin{array}{c} 0.56\\ 0.80 \end{array} \end{array} \end{array}\right]=\left[\begin{array}{c} \begin{array}{c} DN_{0.03}\\ \begin{array}{c} DN_{0.12}\\ DN_{0.24} \end{array} \end{array}\\ \begin{array}{c} DN_{0.36}\\ \begin{array}{c} DN_{0.56}\\ DN_{0.80} \end{array} \end{array} \end{array}\right]\times Gain_{\lambda }+Offset_{\lambda }$$

### Statistical Analysis and Vegetation Index Calculations

Data analysis in this study was conducted using the IBM SPSS Statistics (Statistical Product and Service Solutions 22.0, IBM, Armonk, NY, United States). Graphs were made in the GraphPad software (Version 5.0., Harvey Motulsky and Arthur Christopoulos, San Diego, CA, United States). Statistical evaluation of the N%, SPAD, and LAI datasets was performed according to standard requirements and was shown to be normally distributed. Correlation and regression analysis were conducted, and Pearson’s correlation coefficient (*r*) has been reported as the result of the correlation analysis. Adjusted R squared (*R*^2^) and *p*-values were analyzed and compared by following the standard analysis instructions for all regression analyses.

The VIs were shown to be related to plant growth conditions or the efficiency of photosynthesis. For example, NDRE was closely related to plant chlorophyll content and was used as a parameter to evaluate photosynthesis in plants (Gitelson and Merzlyak, 1994; Gitelson et al., 2003; Duan et al., 2019a,b). Other VIs, such as NDVI, CI_rededge_, CI_green_, and NDGI, were also tested in this study. All five VIs are listed (Table 2).

**TABLE 2:** The details of the vegetation indices used in this study.

Vegetation indices	Formula	References
Normalized Difference Vegetation Index (NDVI)	(ρ800 – ρ670)/(ρ800 + ρ670)	Rouse et al., 1974
Normalized Difference Red Edge Index (NDRE)	(ρ800 – ρ720)/(ρ800 + ρ720)	Gitelson et al., 2003
Red Edge Chlorophyll Index (CI_rededge_)	ρ800/ρ720 – 1	Gitelson, 2005
Green Chlorophyll Index (CI_green_)	ρ800/ρ550 – 1	Gitelson, 2005
Normalized Difference Greenness Index (NDGI)	(ρ800 – ρ570)/(ρ800 + ρ570)	Klem, 2008

### Methodology and Definition of Terms

**FIGURE 1:**
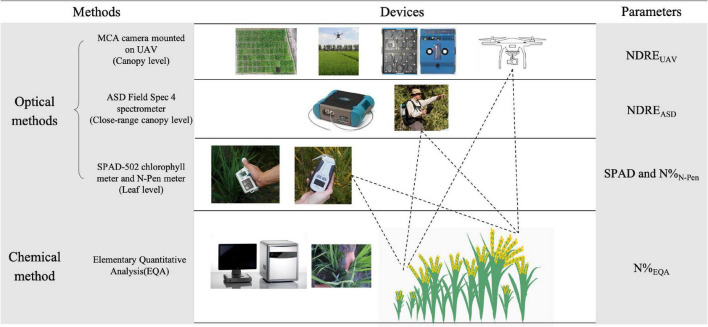
Schematic overview of measured parameters by different methods and devices. NDRE, Normalized Difference Red Edge Index; NDRE_UAV_, NDRE calculated with the canopy reflectance obtained by UAV; NDRE_ASD_, NDRE calculated with the close-range canopy reflectance obtained by ASD; SPAD, SPAD value measured by SPAD-502 chlorophyll meter; N%_N–Pen_, nitrogen content measured by N-pen meter; N%_EQA_, nitrogen content measured by elementary quantitative analysis (EQA).

The internal N of plants was measured by the chemical quantification method (EQA). EQA measurements were used as the reference to evaluate all the RS optical methods and denoted by N%_EQA_ in this study. For N estimation in leaves by RS, SPAD and N-pen were the optical methods used, and they were denoted by SPAD and N%_N–Pen_ in this study. For N estimation in the close-range canopy by RS (1 m above the canopy), ASD was the optical method used and denoted by NDRE_ASD_ in this study. For N estimation in the canopy by RS (50 and 200 m above the canopy, depending on the experimental plot size), MCA mounted on the UAV-RSP was the optical method used and denoted by NDRE_UAV_ in this study (Figure 1).

## Results

### LY9348 Was Identified as a High Nitrogen Use Efficiency Rice Variety With High NUpE and NUtE

**FIGURE 2:**
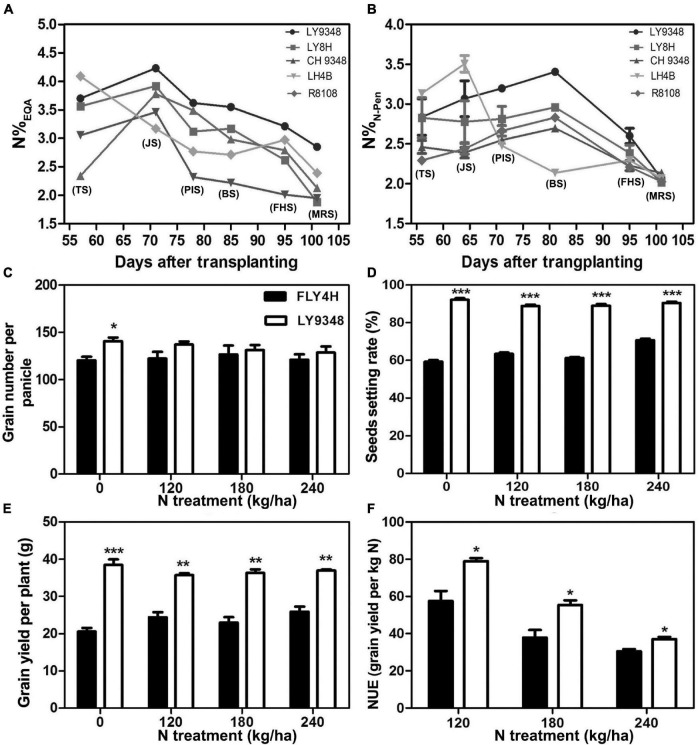
The nitrogen use efficiency analysis of the rice variety LY9348. The nitrogen content was measured by **(A)** EQA (N%_EQA_) and **(B)** N-pen meter (N%_N–Pen_) of different rice varieties. The difference between FLY4H and LY9348 in **(C)** grain number per panicle, **(D)** seed setting rate, **(E)** grain yield per plant, and **(F)** nitrogen use efficiency (NUE) for different nitrogen treatments (N treatment) (*n* = 30). Asterisks denote statistically significant differences between FLY4H and LY9348 (*P < 0.05; **P < 0.01; ***P < 0.001) analyzed by Student’s *t*-test. Rice varieties: LY9348 (circle); LY8H (square); CH9348 (upright triangle); LH4B (inverted triangle); and R8108 (diamond). EQA: elementary quantitative analysis. TS, tillering stage; JS, jointing stage; PIS, panicle initiation stage; BS, booting stage; FHS, full heading stage; MRS, milk ripen stage.

The total growth duration (GD) was divided into six key phenotypic stages as TS, JS, PIS, BS, FHS, and MRS. Compared to its parents (LH4B, CH9348) and the other two rice varieties (R8108 and LY8H), N accumulation in the leaves of LY9348 was higher from JS to the end of MRS, as shown by the EQA analysis (Figure 2A). Especially lower N levels were detected in both the male and female parents of LY9348 for the five growth stages, which suggested that the higher N accumulation ability of LY9348 was not inherited from the parents but was due to the hybrid vigor. The higher nitrogen accumulation in the shoot of LY9348 proved that it had higher NUpE since the five varieties were grown in the same field under the same fertilizer management.

To determine whether LY9348 was a high NUE rice variety with high NUtE, the nitrogen field dosage experiment was conducted. At 0 kg/ha N treatment, effective panicle number (EPN) was 9.2 per plant in FLY4H, and 10.3 per plant in LY9348 (EPN was a critical parameter since rice initiated multiple tillers but not all tillers could produce grains), and panicle length (PL) was 20.2 cm in FLY4H (CK) and 20.8 cm in LY9348. No significant differences in EPN and PL between FLY4H and LY9348 were found, but the thousand grains weight (TGW) of FLY4H (31.24 g) was higher than that of LY9348 (28.94 g). However, the GY of LY9348 was significantly higher than that of FLY4H (9,427.37 vs. 5,687.81 kg/ha) for the 0 kg/ha N treatment (Table 3). Moreover, compared to FLY4H, the grain number per panicle (GNP, Figure 2C, 140.6 vs. 120.0), seed setting rate (SSR, Figure 2D, 92.1 vs. 56.3%) per panicle, and grain yield per plant (GYP, Figure 2E, 38.5 vs. 20.6) of LY9348 were higher than those of FLY4H in the 0 kg/ha N treatment and in the 120, 180, and 240 kg/ha N treatments. Grain yielding per kg N, an evaluation parameter for NUtE, indicated that LY9348 produced higher yield at lower but not higher N treatments (Figure 2F). Therefore, LY9348 was a variety with higher NUtE than FLY4H.

**TABLE 3:** The yield traits of FLY4H and LY9348 for different nitrogen treatments.

N treatment (kg/ha)	Varieties	PL (cm)	EPN	TGW (g)	GY (kg/ha)
0	FLY4H	20.16 ± 0.50	9.22 ± 0.65	31.24 ± 0.17	5687.81 ± 455.23
	LY9348	20.83 ± 0.32	10.33 ± 0.59	28.94 ± 0.01*	9427.37 ± 339.41*
120	FLY4H	19.65 ± 0.21	10.50 ± 0.29	30.08 ± 0.29	6899.36 ± 907.66
	LY9348	20.70 ± 0.10*	10.37 ± 0.46	28.25 ± 0.69*	9468.34 ± 276.28*
180	FLY4H	19.90 ± 0.03	9.90 ± 0.50	30.45 ± 1.11	6813.30 ± 1041.14
	LY9348	20.67 ± 0.25*	10.90 ± 0.40*	28.54 ± 0.09*	9972.17 ± 617.55*
240	FLY4H	19.91 ± 0.12	10.09 ± 1.55	30.41 ± 0.43	7323.83 ± 395.71
	LY9348	20.24 ± 0.56	11.33 ± 0.95	28.40 ± 0.40*	8894.49 ± 388.84*

N treatment: nitrogen treatments include 0, 120, 180, and 240 kg/ha. Values were shown by mean ± standard deviation (n = 30), ha: hectare (1 ha = 10000 m^2^). Statistically significant differences between FLY4H and LY9348 (*P < 0.05) were analyzed by Student’s t-test and labeled with Asterisks.

In conclusion, LY9348 was a high NUE rice variety, with high NUpE and NUtE.

### N Estimation by the SPAD-502 Chlorophyll Meter and the N-pen Meter at the Leaf Level

**FIGURE 3:**
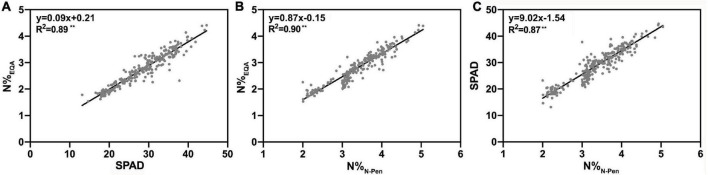
The relationship of **(A)** the nitrogen content measured by EQA (N%_EQA_) and Soil Plant Analysis Development (SPAD) value measured by the SPAD-502 chlorophyll meter (SPAD), **(B)** the nitrogen content measured by EQA (N%_EQA_) measured by the N-pen meter (N%_N–Pen_), and **(C)** SPAD and N%_N–Pen_; **P < 0.01.

To determine the differences in N content estimation between the SPAD-502 chlorophyll meter and the N-pen meter, N%_EQA_ was used to verify the N estimation accuracy at the leaf level. As shown in Figure 3, SPAD and N%_N–Pen_ both exhibited a strong linear relationship with N%_EQA_, with *R*^2^ of 0.89 and 0.90, respectively. This indicated that these two optical meters accurately estimated rice N content during the entire GD. Additionally, a strong linear relationship was also found between SPAD and N%_N–Pen_. It can be inferred that the performance of these two optical meters was similar for N estimation at the leaf level.

However, as shown in Figure 2B, the N-pen meter failed to distinguish N content difference among the five varieties at MRS, and the N%_N–Pen_ values clustered around 2%. To determine the difference in N content estimation at different growth stages, correlation analysis was further conducted between N%_EQA_ and SPAD, and between N%_EQA_ and N%_N–Pen_ at five key stages (Table 4). Generally, SPAD and N%_N–Pen_ showed strong correlations with N%_EQA_ at different growth stages with Pearson’s correlation coefficients above 0.81. Besides, the correlation between SPAD and N%_EQA_ was consistent with the correlation between N%_N–Pen_ and N%_EQA_. Among the five stages, the strongest correlation was found at BS, followed by JS and FHS, and the correlations at PIS and MRS were relatively weak. Pearson’s correlation coefficient between N%_N–Pen_ and N%_EQA_ was 0.81 at MRS, which was the weakest correlation among the five stages, and thus, the N-pen meter failed to distinguish N content difference among the five varieties at MRS (Figure 2). Therefore, the SPAD-502 meter and N-pen meter estimated N content better at BS, JS, and FHS than at PIS and MRS.

**TABLE 4:** Pearson’s correlation coefficients for the correlation analysis between Nitrogen content measured by EQA (N%_EQA_) and Soil Plant Analysis Development (SPAD), and between N%_EQA_ and N-pen meter (N%_N–Pen_).

	JS	PIS	BS	FHS	MRS
SPAD	0.89**	0.81**	0.90**	0.87**	0.87**
N%_N–Pen_	0.91**	0.86**	0.93**	0.90**	0.81**

**Correlation is significant at the 0.01 level (two-tailed).

### N Estimation Using the Analytical Spectral Devices Spectrometer at the Close-Range Canopy Level

Previous studies suggested that NDRE performed the best in estimating the N nutrition parameters among all the VI candidates (Zheng et al., 2020). To test the accuracy of N estimation at the close-range canopy level, ASD was used to estimate N levels in the six key stages during the entire GD to determine the best growth stage by NDRE. Pearson’s correlation analysis was conducted among the 51 rice varieties (Table 5). The correlation between NDRE_ASD_ and SPAD was generally better than that between NDRE_ASD_ and N%_N–Pen_ for all stages analyzed. In BS, NDRE_ASD_ showed the weakest correlation with both SPAD (*r* = 0.28) and N%_N–Pen_ (*r* = 0.01). The correlation between SPAD and NDRE_ASD_ in MRS (*r* = 0.68) and JS (*r* = 0.68) was better than in PIS (*r* = 0.52) and TS (*r* = 0.40). In addition, N%_N–Pen_ showed weak correlations with NDRE_ASD_ (*r* < 0.5) in all stages besides FHS (*r* = 0.70) and MRS (*r* = 0.61). Thus, FHS was identified as the stage that showed the strongest correlation with both SPAD (*r* = 0.81) and N%_N–Pen_ (*r* = 0.70). FHS was the stage when rice plants developed morphological structures, biomass was fundamentally built, and the NDRE_ASD_ estimations were comparably stable. Rapid morphological changes in vegetative development in TS and JS, panicle and inflorescence initiations in PIS and BS, along with canopy and grain color changes in MRS, disturbed the reflectance data during the VI analysis at the close-distance canopy level.

**TABLE 5:** Pearson’s correlation coefficients for the correlation of Normalized Difference Red Edge (NDRE) index (based on ASD) with SPAD and N%_N–Pen_ at different growth stages.

	TS	JS	PIS	BS	FHS	MRS
SPAD	0.40	0.68**	0.52**	0.28	0.81**	0.68**
N%_N–Pen_	0.25	0.17	0.46	0.01	0.70**	0.61**

**Correlation is significant at the 0.01 level (two-tailed).

### Growth Duration Influenced N Estimation Accuracy

**FIGURE 4:**
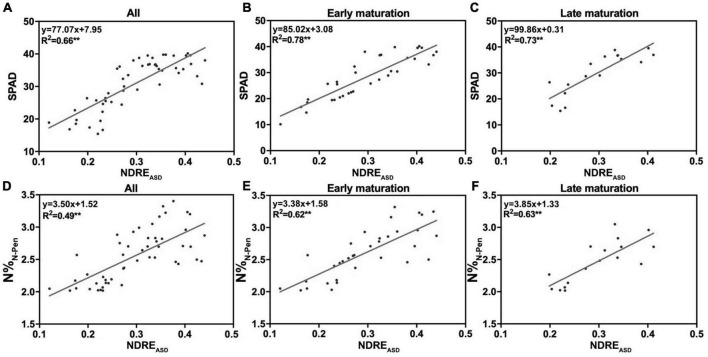
The relationship of NDRE_ASD_ with SPAD and N%_N–Pen_. The relationship of NDRE_ASD_ with SPAD for **(A)** all varieties, **(B)** early maturation varieties, and **(C)** late maturation varieties. The relationship of NDRE_ASD_ with N%_N–Pen_ for **(D)** all varieties, **(E)** early maturation varieties, and **(F)** late maturation varieties; **P < 0.01.

Growth duration is known to influence plant growth, biomass accumulation, flowering time, and yield (Li F. et al., 2018; Liu et al., 2020; Won et al., 2020). Therefore, to test the impacts of GD on N accumulation, 51 rice varieties with GD variations were used. Longer GD was a criterion to separate middle-season rice from early-season and late-season rice in breeding and agricultural practices. Middle-season rice usually grew for more than 100 days, while the others grew for less than 90 days. Thus, 100 days from transplanting to the maturation stage was set as the selection cut-off value to separate 51 rice varieties into two groups: early maturation varieties (EM) with shorter GD and late maturation varieties (LM) with longer GD (Supplementary Table 1). To determine whether GD influenced the correlation of NDRE_ASD_ with SPAD and N%_N–Pen_, NDRE_ASD_ from FHS was determined by linear regression analysis. For each group, *R*^2^ increased as expected. The *R*^2^ between NDRE_ASD_ and SPAD increased from 0.66 (mixed 51 varieties, Figure 4A) to 0.78 (EM, Figure 4B) and 0.73 (LM, Figure 4C). The *R*^2^ between NDRE_ASD_ and N%_N–Pen_ also improved considerably from 0.49 (mixed 51 varieties, Figure 4D) to 0.62 (EM, Figure 4E) and 0.63 (LM, Figure 4F). The improved relationships indicated that the shorter or longer maturation duration of rice varieties influenced the accuracy of estimation of SPAD and N%_N–Pen_ by reflectance features.

### N Estimation by Multiple Camera Array Mounted on Unmanned Aerial Vehicle Remote Sensing Platform at the Canopy Level

To determine the relationship between rice N accumulation and spectral features captured by MCA mounted on UAV-RSP at the canopy level, correlation analysis of 51 rice varieties was conducted between N%_EQA_ (chemical measured N%) and different VIs, such as CI_green_, CI_rededge_, NDRE, NDVI, and NDGI, across the entire GD (Table 6). Spectral features for calculating VIs were extracted from the canopy reflectance at different wavelength bands (550, 570, 670, 720, and 800 nm). Overall, the results showed that all VIs had positive correlations with nitrogen (*r*-value was between 0.25 and 0.80). However, for each VI tested, the correlations with N%_EQA_ showed considerable differences. NDRE showed the strongest correlation (*r* = 0.80), and CI_green_ showed the weakest correlation (*r* = 0.25). The correlation between N%_EQA_ and the different VIs decreased from CI_rededge_ (*r* = 0.78) and NDGI (*r* = 0.76) to NDVI (*r* = 0.74). Therefore, NDRE was selected as the optimal VI to estimate N in rice at the canopy level by MCA mounted on UAV-RSP. Interestingly, in previous studies, LNA and PNA also exhibited better linear relationships with NDRE (*R*^2^ = 0.74 and 0.68) in rice by UAV multispectral imagery (Zheng et al., 2020), which is consistent with the above conclusion.

**TABLE 6:** Pearson’s correlation coefficients for the correlation analysis between N%_EQA_ and different vegetation indices across the entire growth duration (GD).

	CI_green_	CI_rededge_	NDRE	NDVI	NDGI
N%_EQA_	0.25**	0.78**	0.80**	0.74**	0.76**

**Correlation is significant at the 0.01 level (two-tailed).

### N Estimation for the Entire Growth Duration Using Unmanned Aerial Vehicle Remote Sensing Platform

**FIGURE 5:**
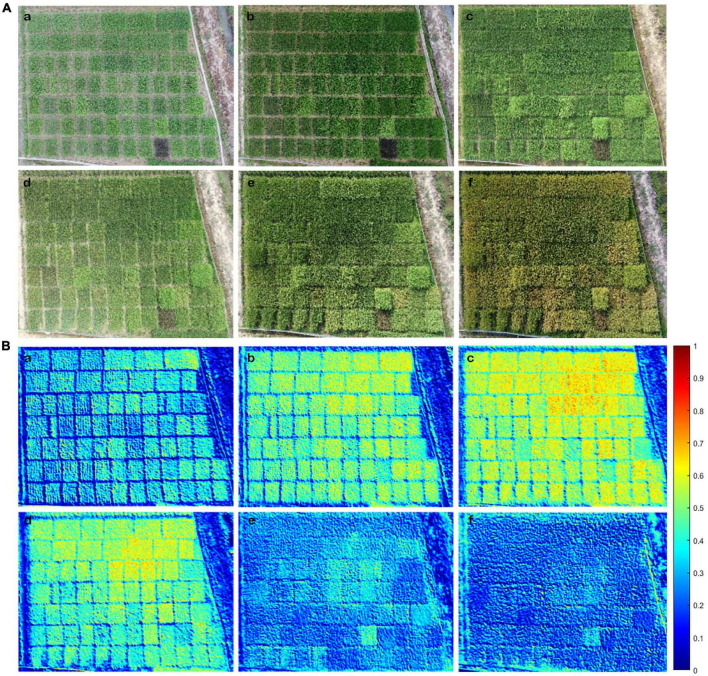
Shown are the RGB image and NDRE_UAV_ that were obtained by the Unmanned Aerial Vehicle Remote Sensing Platform (UAV-RSP) in the six key developmental stages. **(A)** RGB image: **(a)** TS; **(b)** JS; **(c)** PIS; **(d)** BS; **(e)** FHS; **(f)** MRS. **(B)** NDRE_UAV_ illustration: **(a)** TS; **(b)** JS; **(c)** PIS; **(d)** BS; **(e)** FHS; **(f)** MRS. NDRE_UAV_ value varied between 0: cold blue color and 1: warm red color; warmer color indicates higher N accumulations than colder color in rice plants.

To determine whether NDRE_UAV_-assisted N estimation could be applied to the entire GD from transplanting to the harvesting stage, image data of 51 rice varieties were analyzed. RGB image (Figure 5A) and NDRE_UAV_ illustration (Figure 5B) were displayed for the six growth stages. One purple rice variety (Supplementary Table 1: #9 MA LAI Hong) was included in the test group as an internal control since it contained more anthocyanin than chlorophyll, which was expected to be different from the other varieties for reflectance features during data processing. During the GD of all the varieties, the NDRE_UAV_ value increased gradually from TS and JS to PIS and decreased sharply after BS. The minimum and maximum NDRE_UAV_ values for the 51 rice varieties at the different developmental stages were as follows: TS (0.41–0.55), JS (0.46–0.62), PIS (0.38–0.58), BS (0.35–0.54), FHS (0.19–0.41), and MRS (0.15–0.33). Among them, the peak NDRE value was observed in variety #33 (Qingtai Ai) in TS, JS, PIS, and BS and variety #1 (LY9348) in FHS and MRS. Higher values (above 0.5) were observed in JS, PIS, and BS for all varieties, which was associated with rice development, since JS was the stage for rapid biomass increment during vegetative growth and PIS/BS was the transition stage from vegetative growth to reproduction growth. In these stages, more energy was needed for the growth of leaves and stems, along with the production of flowers and seeds later. The lowest NDRE_UAV_ value was observed in the five varieties as #17 (ARC11777, TS), #4 (LH4B, JS, and PIS), #16 (MA MA GU, BS), #7 (ZUIHOU, FHS), and #28 (MO MI, MRS). However, the exact maximum NDRE_UAV_ value, the time to reach and drop from the maximum NDRE_UAV_ value varied across these 51 varieties in each stage or each variety at different stages, indicating that N accumulation variations across developmental stages and different rice varieties were captured and illustrated by NDRE_UAV_. Thus, NDRE_UAV_ was applicable for the evaluation of N accumulation for the rice varieties through the entire GD.

### Model I Was Constructed to Determine the Relationship Between NDRE_UAV_ and N%_EQA_

**FIGURE 6:**
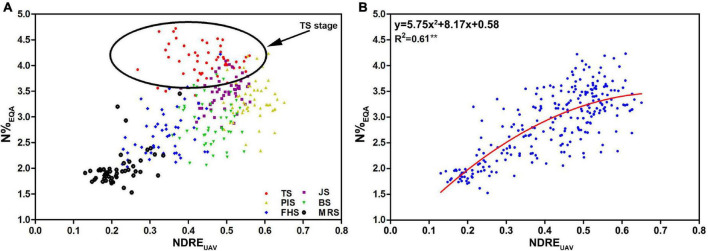
The data plots and regression Model I constructed from 51 rice varieties were based on the UAV-RSP for the six developmental stages. **(A)** Relationship between N%_EQA_ and NDRE_UAV_ (*n* = 306): TS (red); JS (purple); PIS (yellow); BS (green); FHS (blue), and MRS (black); **(B)** regression model of N%_EQA_ and NDRE_UAV_ for the five stages (*R*^2^ = 0.61, *n* = 255; **P < 0.01). The circle in **(A)** highlights the data points of TS.

To determine the accuracy of N estimation by NDRE_UAV_ using UAV-RSP, correlation analysis between NDRE_UAV_ and N%_EQA_ was conducted in different growth stages; 306 data points of the 51 rice varieties from six growth periods were analyzed (Figure 6A). UAV fly height of 50 m was enough to cover each plot (60 plants per plot). After transplanting, rice plants developed from a small size (40 cm height, 5–6 leaves) to a large size (120 cm height, 16–18 leaves). Since biomass changes and canopy modifications were due to nitrogen accumulation, the value should increase from seedling to maturation stages as TS < JS/PIS/BS. After maturation, chlorophyll decomposition and leaf senescence should cause N reduction to a low level from FHS to MRS. In the scattered plot, it was observed that nitrogen content had a high correlation with NDRE_UAV_ from JS, PIS to BS, but a lower correlation from FHS to MRS, as expected. However, the values in TS were unexpectedly higher than in all the other stages. Considering the low biomass, narrow leaves, and small plant size at this stage, the uncovered water part, which exhibited low reflectance in all wavelength bands, might have increased the NDRE_UAV_ amount and overestimated the value. Therefore, data from TS was removed for nitrogen estimation, and the data after JS, when the plant canopy fully covered the water in the field, was processed to reduce data overestimation by NDRE_UAV_.

By removing the TS data, a non-linear regression model was constructed for the relationship between N%_EQA_ and NDRE_UAV_. A stronger relationship between NDRE_UAV_ and N%_EQA_ was shown with *R*^2^ = 0.61 (Figure 6B). Since the N%_EQA_ was measured from individual leaf samples of the 51 rice varieties in the five key growth stages, the regression model (*y* = 5.75 × ^2^ + 8.17x + 0.58) was considered as an RS estimation model for CNC based on actual measurements and set as Model I.

### Model II Was Constructed to Determine the Relationship Between NDRE_UAV_ and N%_EQA_ × LAI

**FIGURE 7:**
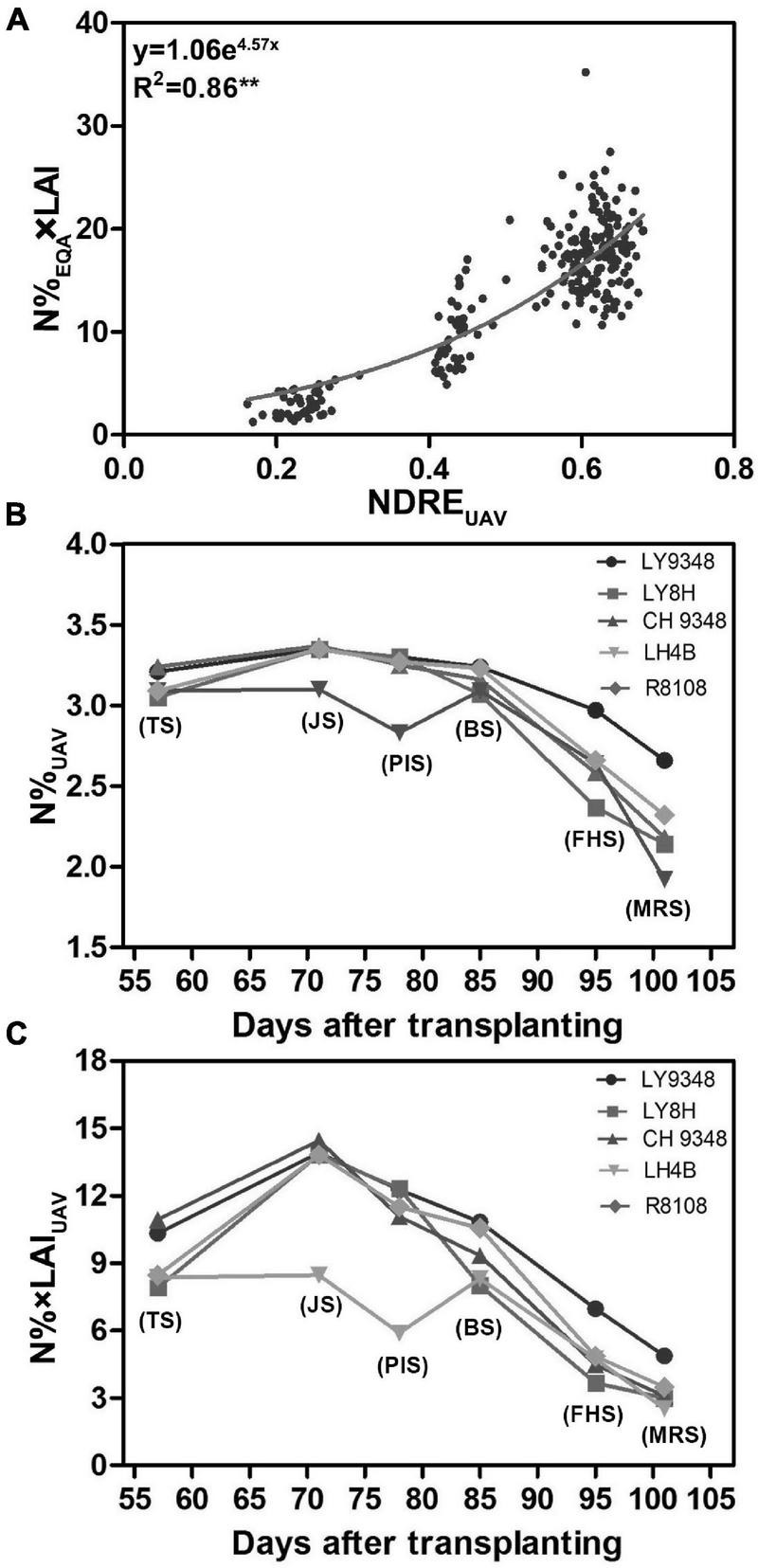
Model II was constructed from 42 rice varieties, and comparisons were performed between Models I and II for nitrogen (N) estimation in LY9348. **(A)** Non-linear regression model constructed between N%_EQA_ × LAI and NDRE_UAV_ during the entire growth period based on the UAV-RSP (*R*^2^ = 0.86, *n* = 252; **P < 0.01); **(B)** N% based on UAV data (N%_UAV_) estimated by Model I (*y* = 5.75^2^ + 8.17x + 0.58, *x*: NDRE_UAV_; *y*: N%_UAV_); **(C)** N% × LAI based on UAV data (N% × LAI_UAV_) estimated by Model II (*y* = 1.06e^4^.^57x^, *x*: NDRE_UAV_; *y*: N% × LAI_UAV_). Each data point in **(B,C)** are the six developmental stages (TS, JS, PIS, BS, FHS, and MRS).

To determine whether the canopy structure could influence the correlation between nitrogen and NDRE_UAV_ in the different growth stages, a training dataset of 42 rice varieties (Supplementary Table 2), which were different from the 51 varieties (Supplementary Table 1) tested above, was used for the analysis. The experimental plot for each variety was larger, containing 1,500 plants in each plot. Therefore, UAV-RSP data collection was performed at 200 m above the canopy to guarantee image collection. LAI and N%_EQA_ were measured from the collected leaf samples, and NDRE_UAV_ was calculated. To monitor the canopy structure in the model, instead of using N%_EQA_, N%_EQA_ × LAI was calculated as the parameter for the analysis. A non-linear model was constructed, and the evaluated regression coefficient *R*^2^ was 0.86 (Figure 7A). The correlation was stronger than that in Model I (*R*^2^ = 0.61). Therefore, by taking the canopy structure, such as LAI, into consideration, nitrogen estimations by NDRE_UAV_ improved, and the regression model (*y* = 1.06e^4^.^57x^) was set as Model II.

To determine whether the constructed Models I and II could be used to detect N related phenotypes, N accumulations of LY9348 variety along with its male and female parents and two other rice varieties, i.e., R8108 and LY8H, were simulated by NDRE_UAV_ estimation through Model I (Figure 7B) and Model II (Figure 7C), respectively. In both model estimations, the same pattern was found, i.e., LY9348 accumulated a higher N level than the other four rice varieties from PIS, BS, FHS, to MRS (Figures 7B,C), which was consistent with the previous measurements (Figures 2A,B). However, in Models I and II, nitrogen values were modified from TS to BS, which was similar for LY9348 and the other varieties, as shown in the N%_EQA_ (Figure 2A) and N%_N–Pen_ (Figure 2B) analysis for these four early developmental stages. However, higher N levels in FHS and MRS, which were associated with the high NUE phenotype of LY9348, could still be detected in Model I (Figure 7B) and Model II (Figure 7C) but could not be detected by the N-pen meter (Figure 2B). Therefore, Model I and Model II were more sensitive than the N-pen meter method in capturing the high NUE phenotype of LY9348 at later stages in the field, although the actual values obtained from each model were different.

### Comparisons Between Model I and Model II for Identification of the High Nitrogen Use Efficiency Phenotype

**FIGURE 8:**
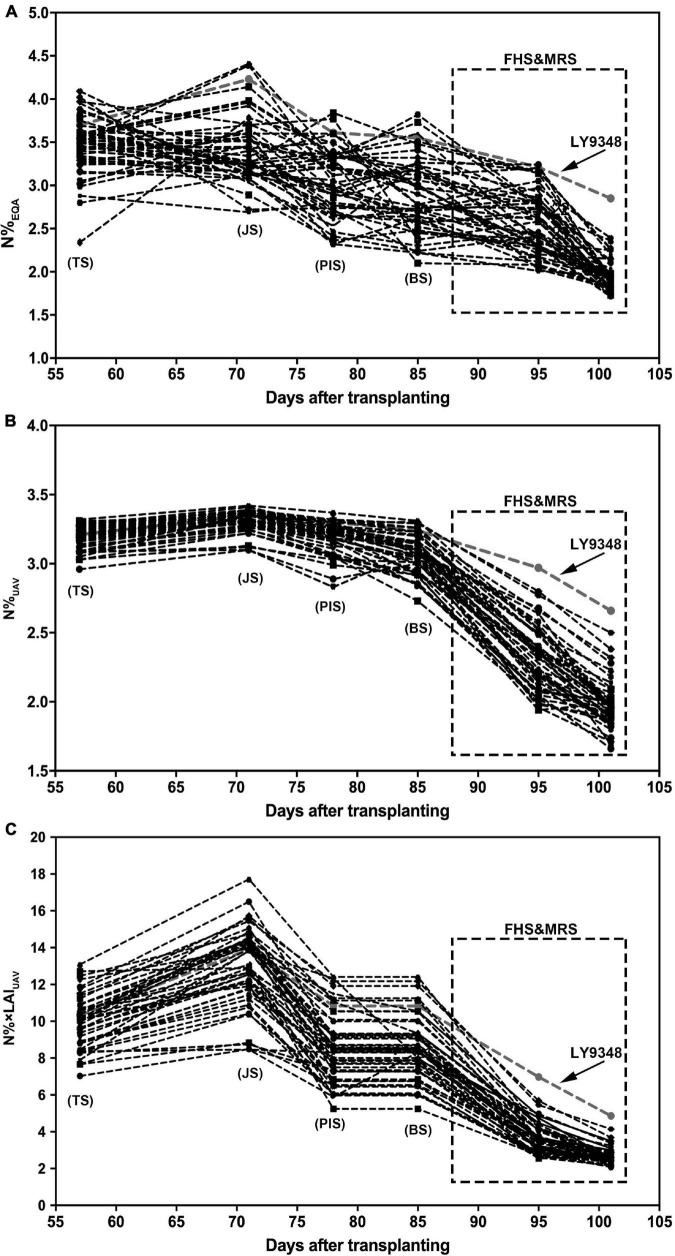
The nitrogen accumulation dynamic curve of 51 rice varieties through the entire growth duration by actual measurement (N%_EQA_, **A**) and estimation by Model I (N%_UAV_, **B**) and Model II (N% × LAI_UAV_, **C**). Model I: *y* = 5.75 × ^2^ + 8.17x + 0.58, *x*: NDRE_UAV_; *y*: N%_UAV_. Model II: *y* = 1.06e^4^.^57x^, *x*: NDRE_UAV_; *y*: N% × LAI_UAV_. FHS and MRS are highlighted by the square regions.

To test the performance and accuracy of Models I and II in larger population selections for the characterization of high NUE phenotypes, data of 51 rice varieties from six growth stages were used for the simulation. The N%_EQA_ of LY9348 showed a median high N level in all stages for the 51 varieties but stayed at the top only in MRS (Figure 8A). Both Models I and II detected the highest nitrogen level in FHS and MRS (Figures 8B,C). However, the N estimation curves across the different stages of the 51 rice varieties from TS, JS, PIS, to BS differed between the two models. Particularly, the line was flattened in Model I but fluctuated in Model II. Since the canopy structure rapidly changed from TS to BS, the sensitivity of Model II, after considering LAI, captured the changes. Therefore, the detection sensitivity and accuracy of Model II were better than those of Model I.

**FIGURE 9:**
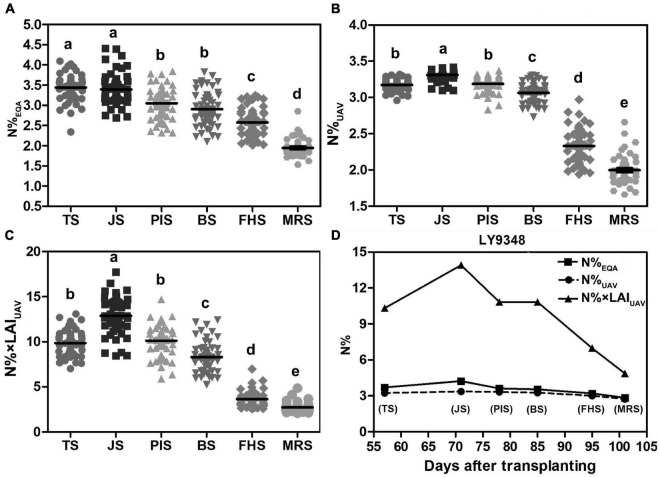
High Nitrogen Use Efficiency (NUE) phenotype determined in LY9348. **(A)** Statistical box plot of N% by actual measurement (N%_EQA_) in 51 rice varieties for the six developmental stages; **(B)** statistical box plot of N% estimated by Model I (N%_UAV_) in 51 rice varieties for the six developmental stages; **(C)** statistical box plot of N% estimated by Model II (N% × LAI_UAV_) in 51 rice varieties in the six developmental stages; **(D)** nitrogen accumulation dynamic curve of LY9348 by actual measurement (N%_EQA_), and estimation by Model I (N%_UAV_) and Model II (N% × LAI_UAV)_: N%_EQA_ (square), N%_UAV_ (circular), and N% × LAI_UAV_ (triangle). The black line in **(B–D)** represents data median, and different letters indicate significant differences (*P* < 0.05, one-way ANOVA, Tukey’s HSD test).

Box plots showed that N%_EQA_ was scattered randomly around the median for all stages checked (Figure 9A). However, the N% estimated by Model I showed a tighter distribution around the median in TS, JS, PIS, and BS compared to that in FHS and MRS (Figure 9B). On the contrary, the N% estimated by Model II showed a less tight distribution around the median in TS, JS, PIS, and BS compared to that in FHS and MRS (Figure 9C).

To determine which model was suitable for high NUE phenotype detection, especially in high-throughput scale for high NUE phenotype identification, the N%_EQA_ and the N level estimated by UAV-RSP data across the whole GD of LY9348 were presented in one figure (Figure 9D). The line of N%_UAV_ in Model I was similar to the line obtained from the EQA measurement in all stages except in JS, where a lower estimation was observed. The N% × LAI_UAV_ estimation by Model II had a value two to four times higher than the value in Model I, and the fluctuation among stages was also greater in Model II than the fluctuation in Model I and that found in the EQA measurement. Both models were capable of determining high N accumulation levels in the LY9348 variety, especially in FHS and MRS, which were specifically associated with the high NUE phenotype of LY9348 (Figure 9D). Therefore, both models could identify the high NUE phenotype.

## Discussion

### High N Accumulation in Full Heading Stage and Milk Ripen Stage Demonstrated the High Nitrogen Use Efficiency Phenotype

This study showed that the high NUE variety LY9348 demonstrated a specific N dynamic curve, which distinguished it from other varieties and its ability to maintain the highest N level after the complete emergence of the panicles, as estimated by NDRE_UAV_. Since a higher N accumulation was also detected by leaf EQA measurement, the authenticity of NDRE_UAV_ was validated (Figures 2A, 9A,B). Besides LY9348 and LY8H, 49 rice varieties were selected from the 3000 genome project (Li et al., 2014), representing higher NUE rice variety groups (*indica*, *intermediate*, *aus/boro*) to build regression Model I (Supplementary Table 1) and 38 *indica* and 2 *japonica* rice varieties, different from the above 49 varieties, were selected from breeding programs for agricultural practices in China to construct regression Model II (Supplementary Table 2). Although the genetic backgrounds for model construction were different, the specific higher N accumulation status of LY9348 was detected in both the models at FHS and MRS (Figures 7B,C, 8B,C), which indicated that this phenotype could be traced reliably across different locations (Ezhou, Hubei, 2017 versus Lingshui, Hainan, 2018) and from genetically mixed sources (51 versus 42). Therefore, maintaining a relatively higher level of N after the initiation of the panicles was shown as a representative character, or interpreted as a specific phenotype, for the high NUE rice variety LY9348.

Many previous studies had shown that an increase in the N supplies at the panicle and grain filling stages, either by medium level application of exogenous soil N, top-dressed N fertilizer, or efficient N remobilization from senescent organs, could modify panicle density type and significantly increased the filled grain numbers, the seed setting rate, and the weight of the grains (Sikder and Gupta, 1976; Wang, 1981; Murty and Murty, 1982; Mohapatra et al., 1993, 2011; Tang et al., 2011; Li et al., 2012; Siebenmorgen et al., 2013; Yoneyama et al., 2016; Wang B. et al., 2018), which was consistent with our results that LY9348 generated more grain numbers, increased the seed setting rate by 20%, and doubled the grain yield per plant in the 0 kg/ha N treatment field trials compared to the results of FLY4H (Figures 2C–E and Table 3), which is a standard high yield CK from the National Rice Industrial Technology System for rice variety certification authorization in China. Besides, excess N supply at the flowering and grain filling stages was also critical for functional leaves, such as the flag leaf, to stay green, to maintain the stability of photosynthetic enzymes, and to guarantee energy supply for improving grain quality and increasing grain weight (Evans, 1989; Thomas and Howarth, 2000; Tang et al., 2005; Dawson et al., 2008; Zhang et al., 2010; Wang et al., 2014; Gu et al., 2017; Luo et al., 2018; Woo et al., 2019). Therefore, a high level of N accumulation in the maturation stages was identified as a high NUE phenotype of rice.

### Moderate-High N Accumulation Level in Vegetative Developmental Stages Was an Important Part of the High Nitrogen Use Efficiency Phenotype

Although the obvious N accumulation differences between LY9348 and other varieties were observed in the panicle and grain developmental stages, the N level in the earlier stages was also important. Many previous studies concluded that N fertilization added at TS and BS were effective in increasing tiller numbers, biomass, and photosynthesis products. However, over-application of N in these stages initiated more ineffective tillers, shallow roots, unhealthy shoot morphological structures, and delayed transition from vegetative to reproductive growth, causing a greater loss in yield (Pan et al., 2011; Andrew et al., 2013; Sui et al., 2013; Hu and Zhang, 2014; Zhu et al., 2016). Moreover, proper N addition at PIS and BS increased the number of panicles, spikelets per panicle, the seed setting rate, and grain weight. However, these yield-related traits decreased when too much N was applied. Compared to the NDRE_UAV_ value among the 51 rice varieties in TS (0.41–0.55), JS (0.46–0.62), PIS (0.38–0.58), and BS (0.35–0.54), the LY9348 variety displayed a relatively higher, but not the highest, NDRE_UAV_ value in TS (0.50), JS (0.56), PIS (0.54), and BS (0.51). Variety #33 (Qingtai Ai) showed the highest NDRE_UAV_ value in all the four stages but was not a high NUE variety. Therefore, control N accumulation at a moderate-high level during the vegetative and transition developmental stages should be considered to be a part of the high NUE phenotype for overall evaluation. To determine if the N accumulation phenotypes of LY9348 in all the stages are representative, phenotypic and field analysis should be conducted in the future to test two hypotheses: (1) these phenotypes are true for all high NUE varieties; and (2) a new variety can be inferred to be using N efficiently if its N phenotypes mimic that of LY9348.

### Dynamic N Curve Captured by Unmanned Aerial Vehicle Remote Sensing Platform Was Identified as a New High Nitrogen Use Efficiency Phenotype

Attempts have been made to select high NUE breeding varieties; however, the progress has been slow. Because variation in N levels is found in both the spatial (canopy morphological changes) and temporal (developmental variation through the entire GD) modes, it is hard to capture the changes of N in plants in either mode. The RS technology is the ideal way to determine N fluctuation with low effort and cost, in real-time and non-destructively, and can replace the traditional methods that are tedious and destructive. However, the challenges are to determine what the high NUE phenotypes should be from the perspective of RS and whether the N dynamics demonstrated by RS are reliable and repeatable in the breeding trials.

Accumulation and remobilization of N within plants is a dynamic balance of environmental N supplies, plant N needs, and plant N accumulation and transportation abilities (Han et al., 2015). Phenotypic descriptions of N levels in plants should be analyzed as time-series data, although due to the technical and methodological limitations, it has been analyzed as a snapshot or fragmental dataset by traditional methods. UAV-RSP combines statistics, reflectance, and calculus-based computational data to construct and digitalize N-related temporal biological changes, which allows the dynamic N curve phenotypes to be evaluated in a video-mode view across the entire GD range. This study showed that the high NUE phenotype maintained a moderate-high level of CNC in the earlier developmental stages from TS, JS, PIS to BS and the highest, but gradually decreasing, level of CNC in FHS and MRS. This time-series N dynamic phenotype could be used as a new high NUE phenotype for field screening.

Moreover, LAI increased the accuracy of the analysis of this phenotype in Model II estimation which also suggested that spatial-series data with canopy structure differences could provide more information. The combination of time-series and spatial-series data might better illustrate the accumulation and distribution of N in future studies regarding changes in NUE.

### High Nitrogen Use Efficiency Variety Was the Key Source to Identify High Nitrogen Use Efficiency Related N Accumulation Phenotypes

For breeding purposes, phenotype identification is very important, and the phenotype identified must be traceable. For example, the yield of jasmine rice is low, but high yield rice has no aroma (Usman et al., 2020; Dhondge et al., 2021). If breeders want to create high-yield aroma rice, they need to select aroma rice and high-yield rice as parents and perform crosses and backcrosses for several generations. In each generation, thousands of offspring of each cross must be evaluated for phenotypes of aroma and yield. Since these two phenotypes can either be measured (yield) or tasted (aroma), it is possible to create high-yield aroma rice. However, it is difficult to create high NUE rice varieties because neither NUpE nor NUtE phenotypes are well-defined. In previous studies, NUpE varieties were selected since absorption of N could be quantified, and plant N transporter genes were cloned and analyzed intensely in the past decades. However, evaluating NUtE was challenging because it was hard to quantify how much N, after absorption by plants, was being used to increase the grain yield and improve grain quality, instead of being used to grow bigger leaves or increase biomass. The lack of well-defined traceable phenotypes, especially for NUtE, was the limiting factor for the generation of higher NUE varieties.

High NUE rice germplasm is the key source to identify high NUE phenotypes. However, NUE varieties are rare. Specifically, very few NUtE rice varieties have been reported. The over-application of N for agricultural purposes has a considerable adverse impact on the environment. The rice variety LY9348, which contains both the high NUpE and NUtE phenotypes, showed great efficiency and productivity value and should be studied intensely to provide clues and ideas for future studies regarding increasing NUE. We adopted RS technologies to identify the specific N accumulation phenotype of LY9348 and tried to determine an easier method to accelerate the identification of the high NUE phenotypes.

Through UAV-RS, we identified the specific N accumulation phenotype of LY9348, and this was the first study to show a dynamic N accumulation phenotype across the whole GD in a mixed genetic background (51 and 42 collections). Since LY9348 was unique in NUE, its N accumulation dynamic curve was quite different from that of the other varieties. Therefore, its dynamic N curve phenotype could be highlighted as a single specific curve in different genetic backgrounds across locations and years. This phenotype provided a valuable way to quantify NUE and can be used to select high NUE varieties when LY9348 is used as a high NUE donor, which is promising for the generation of NUE varieties in the future using the UAV-RS method and the established models.

### Enlarged Gene Pools for the Selection of High Nitrogen Use Efficiency Varieties

In traditional NUE selection, breeders usually choose high NUE parents to create higher NUE hybrids. Interestingly, we found that LY9348, as a hybrid rice variety, had high NUE, but its male (CH9348) and female (LH4B) parents had low NUE (Figure 2A). This indicated two important aspects regarding the generation of high NUE varieties: (1) The high NUE phenotype of LY9348 might have been generated by hybrid vigor and not inheritance. Higher NUE varieties in hybrid lines formed through heterosis from lower NUE parental lines were also reported in maize (Wang Z. et al., 2019); (2) High NUE varieties might be created through multiple maternal and paternal combinations, even if the parental varieties do not show high NUE phenotypes. Hence, a larger gene pool can be used for the selection and generation of high NUE varieties.

Shorter or longer GD influenced the accuracy of estimation of SPAD and N-pen of rice varieties at the leaf level. From an agricultural standpoint, variations in biomass accumulation, leaf color, nitrogen remobilization and canopy structure in different varieties were all influenced by GD. Although GD did not affect the high NUE dynamic curve identified in LY9348, for agricultural inspections and phenomics studies, GD should be considered as an important parameter, especially when hundreds or thousands of varieties with different genetic backgrounds need to be evaluated simultaneously.

Moreover, studies should determine if the specific high NUE phenotype can be genetically transferred from LY9348 to its offspring population through backcrosses or crosses with low NUE rice varieties. If the same high NUE phenotype can be traced in the population for segregation, genetic tools such as molecular markers might be identified to assist in the selection process. Phenomics, genomics, and metabolomics in specific phenotypic stages might simultaneously provide more information to understand the high NUE networks along with their mechanisms and regulations.

### Minimum Experimental Requirements for Field Screening

For high-throughput phenomics analysis and large population breeding selections, minimum experimental plot size and minimum individual plants needed for determining a reliable phenotype should be tested to minimize expenses and increase selection efficiency in agricultural practices. Several trials were conducted in Ezhou, Hubei (2017) and Lingshui, Hainan (2018). Plots of 1.2 m × 1.6 m with 60 plants (6 rows and 10 plants/row) were technically sufficient for the NDRE_UAV_ analysis in our experiments for N inspections. Plots smaller than this would have reduced the evaluation accuracy (data not shown). Compared to the traditional 1,500 plants/66.67 m^2^ plot design in Lingshui, Hainan (2018) and 4.5 m × 8 m or 5 m × 6 m plots (Parco et al., 2020) in other nitrogen-related studies, the cost was considerably lower. We also found that a 20 cm space between each plot was helpful in image processing, especially in the later developmental stages when the plant biomass increased dramatically. Therefore, the cultivation of hundreds or even thousands of rice varieties in one small field for N inspection by this method was shown to be possible, which supported the screening of NUE phenotypes in a controlled experimental background.

For UAV flight density, our trial showed that N inspection and data collection every 5–7 days was enough for the entire GD. In the future, collection of data every 7–10 days or even after a longer duration can be tried to establish less laborious but reliable protocols for high-throughput phenomics and effective agricultural management.

### Factors That Influence the Tracing of High Nitrogen Use Efficiency Phenotypes

To resolve the application-related problems of high-throughput phenomics in field trials and make the life of breeders easier, we tested three aspects for determining the effects on the reliable identification of high NUE phenotypes.

(1) Five VIs were compared, and NDRE was relatively better for estimating N contents in rice plants, which was confirmed by the studies of other researchers where the UAV platform was used (Zheng et al., 2020). Besides, we tested different scales of reflectance data such as NDRE_ASD_, using the UAV platform at 50 m (NDRE_UAV50_) and 200 m above (NDRE_UAV200_) the canopy. SPAD, N%_N–Pen_, and N%_EQA_ were also compared. There was a strong linear relationship between N%_N–Pen_ and N%_EQA_ with *R*^2^ above 0.65 in MRS and 0.86 in BS. Interestingly, although N%_N–Pen_ showed a higher correlation with N%_EQA_, it failed to distinguish N% contents of LY9348 from other varieties at FHS and MRS because the saturation of reflectance features measured and estimated by the N-pen meter could not detect N% below 2% (Figure 2B). However, NDRE, calculated by UAV-RSP from different canopy heights (50 m; 200 m), detected the N dynamic phenotypes of LY9348 (Figures 7B,C, 8B,C), which indicated that the flight height did not influence the high NUE phenotype tracing and discrimination in the field trials. From the perspective of effort and expenses, N%_EQA_ > NDRE_ASD_ > NDRE_UAV200_: N%_EQA_ × LAI > N%_N–Pen_ > NDRE_UAV50_: N%_EQA_. Therefore, Model I was more efficient and less expensive for inspecting the high NUE phenotype than Model II, although the sensitivity of Model II was better than Model I. Breeders can choose different methods according to their needs and budgets in the future.
(2) In the estimation of Models I and II, we did not separate leaves and panicles for N estimation and included all the reflectance features captured from our experimental plot of each variety for a mixed N estimation. The results showed that the high NUE phenotype could be detected and easily identified, especially in FHS and MRS. This suggested that the measurements of LNC and CNC separately might not be necessary for the identification of high NUE phenotypes, which simplified data extraction and reduced the complexity of the analysis.
(3) The *indica* rice showed higher NUE ability than the *japonica* rice (Hu et al., 2015). However, N accumulation in LY9348 was even higher than that in the *indica* rice groups, especially in FHS and MRS. This confirmed that the high NUE phenotype we identified was specific and could be traced in mixed genetic backgrounds. This suggested that high NUE phenotypes cannot be masked by N variations among different varieties in large-scale genetic screening. Further studies should test whether the high NUE phenotype can also be detected in mixed *japonica* rice varieties and whether the dynamic N curves vary with lower or higher N treatments.

The N dynamic time-series phenotype identified in LY9348 can assist high NUE phenotype field screening in rice as a screening standard. More high-NUE phenotypes might be captured if other known high NUE varieties are evaluated by this method. Technical improvements could greatly speed up the process of screening for high NUE rice varieties to overcome the long-standing challenge of decreasing the application of N in agricultural practices; thus, reducing costs and environmental damages.

## Conclusion

The specific high NUE phenotype of LY9348 was first identified and illustrated by two models built with NDRE as the selected VI with N%_EQA_ and N%_EQA_ × LAI among two genetically selected rice varieties (*n* = 51, 42) using multispectral data in the paddy field through UAV-RSP. The phenotype was characterized as a N dynamic time-series curve with moderate higher N level from TS till BS but the highest N level maintained after FHS till MRS compared with any other varieties tested. Several impact parameters were evaluated: (1) The identification of the high NUE phenotype was not influenced by the genetic background and flight height of UAV-RSP; (2) Mixed varieties with longer and shorter GD during analysis reduced the strength of the relationship between NDRE_ASD_ and N%_N–Pen_ (to *R*^2^ below 0.5). Hence, it was better to analyze them separately; (3) Reflectance of water in paddy fields caused problems of overestimation. Therefore, tillering stage data was excluded from the model analysis; (4) Canopy structure was a key factor in influencing the sensitivity and accuracy of N measurement by NDRE_UAV_. Thus, N% × LAI was a better parameter to predict N% in large field selections; (5) Minimum field plot size (1.2 m × 1.6 m) and plant sample size (*n* = 60) for valid assessments by the method were determined and were shown to be effective for data extraction and analysis. In the future, not only the identified phenotype can support large or super-large scale high-throughput N-related phenomics analysis and reliably select high NUE varieties to reduce environmental problems caused by N, but also N inspection and high NUE phenotype selections by this method or other platforms explored would be useful for the system construction of smart breeding and precision agriculture.

## Data Availability Statement

The original contributions presented in the study are included in the article/Supplementary Material, further inquiries can be directed to the corresponding author/s.

## Author Contributions

XW initiated the research idea, designed the experiments, and finish the writing of this manuscript. SF and RZ provided the infrastructure for the study site and platform to make this study possible. YP and YG provided important insights and suggestions on this study from the perspective of remote sensing experts. TL did the field experiments and data analysis and part of the writing. BD did the UAV data collection and analysis. XL, YM, and ZY did sample collection and procession in the field. All the authors made significant contributions, read, and approved the final manuscript.

## Conflict of Interest

The authors declare that the research was conducted in the absence of any commercial or financial relationships that could be construed as a potential conflict of interest.

## Publisher’s Note

All claims expressed in this article are solely those of the authors and do not necessarily represent those of their affiliated organizations, or those of the publisher, the editors and the reviewers. Any product that may be evaluated in this article, or claim that may be made by its manufacturer, is not guaranteed or endorsed by the publisher.
