# Supplementary figures and images for: Identification of High Nitrogen Use Efficiency Phenotype in Rice (Oryza sativa L.) Through Entire Growth Duration by Unmanned Aerial Vehicle Multispectral Imagery
Source: Front Plant Sci. 2021 Dec 3;12:740414. doi: 10.3389/fpls.2021.740414 (PMC8678090; doi:10.3389/fpls.2021.740414)

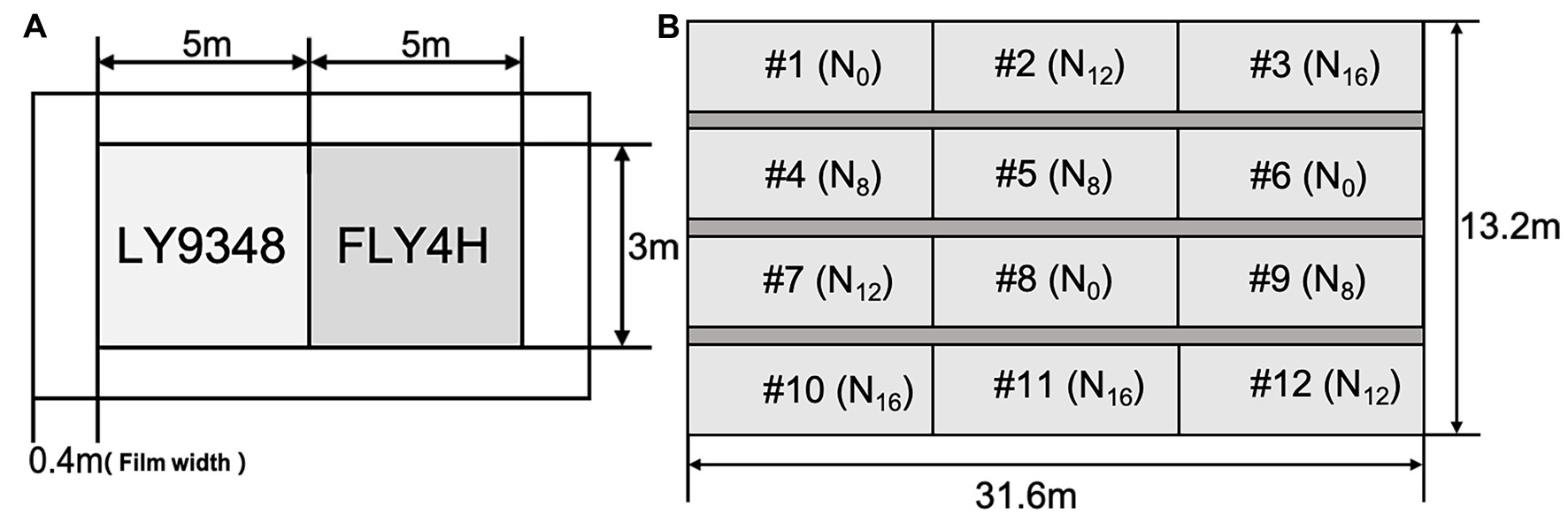

Supplement: Supplementary file 1 [file Image_1.JPEG]
