# Supplementary material for: Identification of High Nitrogen Use Efficiency Phenotype in Rice (Oryza sativa L.) Through Entire Growth Duration by Unmanned Aerial Vehicle Multispectral Imagery
Source: Front Plant Sci. 2021 Dec 3;12:740414. doi: 10.3389/fpls.2021.740414 (PMC8678090; doi:10.3389/fpls.2021.740414)
Supplement: Supplementary file 2 [file Data_Sheet_1.docx]

Supplementary Material

**Supplementary Table 1. The variety information of 51 rice varieties in Experiment 2 and 3.**

| **No.** | **Varieties** | **Durations** | **Origin** | **Varietal Groups** | **No.** | **Varieties** | **Durations** | **Origin** | **Varietal Groups** |
| --- | --- | --- | --- | --- | --- | --- | --- | --- | --- |
| 1 | LY9348 | LM | China | *Indica* | 27 | AI TUO GU 151 | EM | China | *Indica* |
| 2 | LY8H | LM | China | *Indica* | 28 | MO MI | EM | China | *Indica* |
| 3 | CH9348 | LM | China | *Indica* | 29 | E ZI 100 | EM | China | *Indica* |
| 4 | LH4B | EM | China | *Indica* | 30 | CUN GU NUO | EM | China | *Indica* |
| 5 | R8108 | LM | China | *Indica* | 31 | ITA 117 | EM | Nigeria | *Indica* |
| 6 | GX 6 HAO | LM | China | *Indica* | 32 | NCS349 | EM | India | *Indica* |
| 7 | ZUIHOU | LM | Japan | *Indica* | 33 | QING TAI AI | EM | China | *Indica* |
| 8 | IR77298-14-1-2-10 | LM | Philippines | *Indica* | 34 | PL 3165 | EM | China | *Indica* |
| 9 | MA LAI HONG | EM | Malaysia | *Indica* | 35 | HEI DU 4 | EM | China | *Indica* |
| 10 | DIAN RUI 409B | LM | China | *Indica* | 36 | BaWangBian 1 | LM | China | *Intermediate* |
| 11 | 531 | EM | Philippines | *Indica* | 37 | XU GU NUO | EM | China | *Indica* |
| 12 | FACAGRO 406 | EM | Burundi | *Indica* | 38 | IR 19058-107-1 | EM | China | *Indica* |
| 13 | QUN XUAN ZAO | EM | China | *Indica* | 39 | IR 3839-1 | EM | Japan | *Indica* |
| 14 | IRAT 10 | EM | Ivory Coast | *Intermediate* | 40 | Ratnagiri45-2 | LM | Philippines | *Indica* |
| 15 | GL AI 15-1 | EM | China | *Indica* | 41 | PORONG | EM | Malaysia | *Indica* |
| 16 | MA MA GU | EM | China | *Indica* | 42 | TaiChung SenYu214 | LM | China | *Indica* |
| 17 | ARC 11777 | EM | India | *Aus/boro* | 43 | B737G-KN-23-1 | LM | Philippines | *Indica* |
| 18 | FAN WU | EM | China | *Indica* | 44 | IR73571-3B-11-3-K2 | LM | Burundi | *Indica* |
| 19 | E ZI 110 | EM | China | *Indica* | 45 | LUAN DAO | EM | China | *Indica* |
| 20 | XIAO HONG GU | EM | China | *Indica* | 46 | YOU ZHAN | LM | Ivory Coast | *Intermediate* |
| 21 | DANG YU 5 HAO | EM | China | *Indica* | 47 | CHD AI 3 HAO | LM | China | *Indica* |
| 22 | 78 XUAN WU | LM | China | *Indica* | 48 | 88B | EM | China | *Indica* |
| 23 | KUNJUKUNJU | EM | India | *Indica* | 49 | NJ 11 HAO | EM | India | *Aus/boro* |
| 24 | PERUBAK LUEY | EM | Malaysia | *Indica* | 50 | ZX 232 | EM | China | *Indica* |
| 25 | RP20-12 | EM | India | *Indica* | 51 | LS 1 Hao | LM | China | *Indica* |
| 26 | GZ 1368-5-4 | EM | Egypt | *Indica* |  |  |  |  |  |

Durations: entire growth durations; LM: late maturation; EM: early maturation. Varietal Groups: The 3000 rice genomes project classified 3000 rice accessions into five groups (*japonica, indica, aus/boro, basmati/sadri and intermediate*;) (Li et al. 2014).

**Supplementary Table 2. The variety information of 42 rice varieties in Experiment 4.**

| **No.** | **Varieties** | **Institution** | **Varietal Groups** | **No.** | **Varieties** | **Institution** | **Varietal Groups** |
| --- | --- | --- | --- | --- | --- | --- | --- |
| 1 | 23 YOU 418 | JAAS | *Japonica* | 22 | RONG LYHZH | WHU | *Indica* |
| 2 | XD15-90A/XH18 | XNU | *Indica* | 23 | LUO LY4HZH | WHU | *Indica* |
| 3 | ZY 6 HAO | YZU | *Indica* | 24 | YI LYYZH | WHU | *Indica* |
| 4 | 1892S/Z12 | NJAU | *Indica* | 25 | RONG LYYZH | WHU | *Indica* |
| 5 | 288S/Z12 | NJAU | *Indica* | 26 | LUO LY3YZH | WHU | *Indica* |
| 6 | SHY 114 | SHAAS | *Japonica* | 27 | LUO LY 769 | WHU | *Indica* |
| 7 | LY8H | WHU | *Indica* | 28 | LY 13311 | WHU | *Indica* |
| 8 | LY9348 | WHU | *Indica* | 29 | LUO LY 6311 | WHU | *Indica* |
| 9 | HY 3348 | WHU | *Indica* | 30 | LUO LY 8311 | WHU | *Indica* |
| 10 | LHY3615 | WHU | *Indica* | 31 | LUO LY 4311 | WHU | *Indica* |
| 11 | LHY 3618 | WHU | *Indica* | 32 | YI LY 311 | WHU | *Indica* |
| 12 | LHY 8348 | WHU | *Indica* | 33 | RONG LY 311 | WHU | *Indica* |
| 13 | LHY 3338 | WHU | *Indica* | 34 | LUO LY 10311 | WHU | *Indica* |
| 14 | LHY 7348 | WHU | *Indica* | 35 | LUO LY1HZH | WHU | *Indica* |
| 15 | LY1564 | WHU | *Indica* | 36 | LUO LY1YZH | WHU | *Indica* |
| 16 | LHY 2564 | WHU | *Indica* | 37 | LUO LY1311 | WHU | *Indica* |
| 17 | LHY 2615 | WHU | *Indica* | 38 | 203A/S931 | WHU | *Indica* |
| 18 | LHY 7615 | WHU | *Indica* | 39 | 103A/S931 | WHU | *Indica* |
| 19 | LHY 7618 | WHU | *Indica* | 40 | 288S/Z07 | WHU | *Indica* |
| 20 | LY1318 | WHU | *Indica* | 41 | 288S/Z04 | HNYRRC | *Indica* |
| 21 | YI LYHZH | WHU | *Indica* | 42 | 24S/Z04 | NJAU | *Indica* |

JAAS: Jiangsu Academy of Agricultural Sciences, XNU: Xinan University (XNU), YZU: Yangzhou University NJAU: Nanjing Agricultural University, SHAAS: Shanghai Academy of Agricultural Sciences, WHU: Wuhan University (WHU), HNHRRC: Hunan Hybrid Rice Research Center.

**Supplementary Figure legends**

**Supplementary Figure 1. Experimental design for field nitrogen dosage analysis.** A: Block size and design; B: Twelve blocks field design for four nitrogen dosages treatments (N_0_=0 kg/ha, N_8_=120 kg/ha, N_12_=180 kg/ha, N_16_=240 kg/ha).
